# Reliability and validity of the FFQ and feeding index for 7-to 24-month-old children after congenital heart disease surgery

**DOI:** 10.1186/s12887-022-03357-4

**Published:** 2022-06-16

**Authors:** Yiling Lei, Yang Liu, Chunmei Hu, Yanqin Cui, Rui Gao, Xiuxiu Li, Yanna Zhu

**Affiliations:** 1 Department of Maternal and Child Health, School of Public Health, Sun Yat-Sen University, Guangzhou, Guangdong Province, China; 2 Guangzhou Women and Children’s Medical Center, Department of Zhujiang New Town), Guangzhou, Guangdong Province, China; 3 Shenzhen Nanshan District Maternal and Child Healthcare Hospital, ShenzhenGuangdong Province, China; 4 Sun Yat-Sen Global Health Institute, Institute of State Governance, Sun Yat-Sen University, Guangzhou, Guangdong Province, China

**Keywords:** Infants and young children, Food frequency questionnaire, Reliability and validity, Infant and child feeding index, Congenital heart disease

## Abstract

**Background:**

Congenital heart disease (CHD) is the most common congenital defect in neonates. Infants with CHD often have more nutritional difficulties, but currently, there is no unified Food Frequency Questionnaire (FFQ) for infants and young children aged 7–24 months in China. Therefore, we designed this study to assess the reliability and validity of the FFQ and feeding index for 7-to 24-month-old children after congenital heart disease surgery.

**Methods:**

From July to October 2018, infants and young children aged 7–24 months after congenital heart disease surgery in Guangzhou were selected. Participants were categorized into two groups, in the first group (*n* = 95), the FFQ was completed twice at intervals of 7–10 days to assess reproducibility. In the second group (*n* = 98), participants accomplished both the FFQ and the 24-h diet records from 3 consecutive days to assess validity. The score of the Infant and Child Feeding Index (ICFI) and its qualified rate were caculated. Intraclass correlation coefficients (ICC) and Spearman correlation coefficient (SCC) were calculated for reliability and validity, respectively.

**Results:**

The average intraclass correlation coefficients and spearman correlation coefficient of the FFQ were 0.536 and 0.318, all with statistical significance except the frequency of meat added. The ICFI of the first group was 8.61 (± 3.20), the qualified rate was 0.06% (6/95). The intraclass correlation coefficients of the ICFI ranged from 0.374 to 0.958; and the spearman correlation of the ICFI was -0.066 to -0.834.

**Conclusions:**

The FFQ possesses satisfactory reliability and moderate validity. The reliability of the ICFI is acceptable, but the validity results are quite different, indicating that the questionnaire is limited in the evaluation of the ICFI.

**Supplementary Information:**

The online version contains supplementary material available at 10.1186/s12887-022-03357-4.

## Background

Congenital heart disease (CHD) is the most common congenital defect in neonates,with a birth prevalence of approximately 8 per 1,000 worldwide [1, 2]. CHD refers to children aged 0–3 who have anatomical changes or insufficiency in the development of heart and large blood vessels during embryonic development [1, 3, 4]. Infants with CHD often have more nutritional difficulties, including [5] malabsorption, decreased energy intake or increased energy damand caused by infections and/or increased metabolism [4]. So the proportion of infants with CHD who suffer from malnutrition is higher than normal infants [6]. Therefore, after the disease restriction is lifted, reasonable feeding is essential for the postoperative recovery and growth of infants with congential heart disease [6, 7]. However, due to the lack of nutritional status survey methods, there are relatively few studies on the diet of 7–24 months old children after congenital heart disease surgery.

Food frequency questionnaires (FFQs) are the most frequently used tool applied in large-scale epidemiological studies to assess dietary intake, as they assess usual dietary intake over longer periods of time in a relatively short time frame [7–9]. However, the type of food listed in each FFQ, the length of the survey period, the interval frequency, the food share evaluation method, the nutrient database, and the survey method are not identical [10]. In addition,different groups of people have different dietary habits and geographical environment, and the accuracy of dietary intake information is closely related to the reliability and validity of the FFQ in the people who use it [11]. Therefore,FFQ requires adoptions based on people’s dietary habits and geographic location before applying to different population [12, 13]. At present, the FFQs in China are more widely used in adolescents, youth, the elderly or special groups (such as athletes), but less for infants and young children aged 7–24 months [10]. Therefore, it is necessary to design one FFQ for infants and young children aged 7–24 months and test the reliability and validity of it.

The Infant and Child Feeding Index (ICFI) is a comprehensive index to assess the feeding status of infants and young children, and has a certain guiding role in the feeding behavior of caregivers. The concept of the ICFI was first proposed by Ruel and Menon and primarily used for infants and young children between 6–36 months old [14], and they developed a multi-dimensional assessment scale with five indicators, including breastfeeding, bottle feeding or none, type of food, frequency of feeding food, general feeding frequency [15]. Studies have also found that higher scores were associated with lower rate of malnutrition [16].

The aims of the study were to evaluating the reliability and validity of the FFQ for 7–24 months old infants and young children after congenital heart disease, to calculate the scores and the pass rate of the ICFI, and test the reliability and validity of the ICFI.

## Methods

### Study design

The study is an observational study that performed in a convenience sample of 7–24 month-old infants and young children after congenital heart disease surgery from July to October 2018 in the Guangzhou Women and Children Science Center. Participants who were followed up and checked regularly from August to October 2018 in Guangzhou Women's and Children's Medical Center were included. While the individuals with poor postoperative prognosis, combined with other severe wasting diseases, physical deformities, difficulty in feeding, and failure of guardians to complete the survey due to difficulties in understanding or filling will be excluded. This study was approved by the Ethical Committee of School of Public Health at Sun Yat-Sen University. Written informed consent was obtained from all participants.

### Data collection

**Fig. 1:**
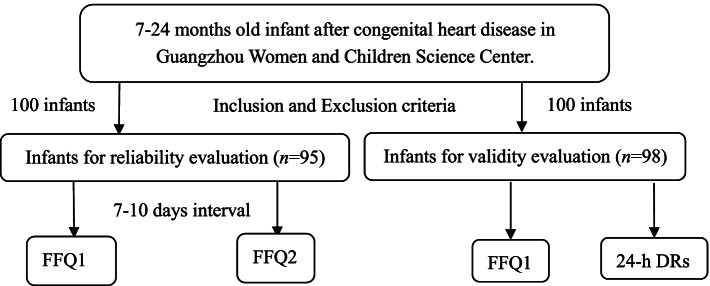
Flow chart of data collection

Face-to-face interviews were conducted by a trained and experienced investigator. The FFQ and the 24-h diet records for 3 consecutive days (24 h DRs) were filled by infants' mother (caregivers). Food mold was used to help participants to estimate portion size. Participants include two parts. In the first part, the FFQ was completed twice at intervals of 7–10 days to assess reproducibility. In the second part, participants accomplished both the FFQ and 24 h DRs at the same time. The method allowing the validity to be assessed (Fig. 1).

The ICFI was introduced and improved with the growth curve of Chinese infants by Jianqiang Lai in 2012 [14, 17, 18], which contains the following terms: breastfeeding or not, feeding with bottles or not, variety and frequency of food in the last 24 h, days of adding complementary food in the past week, the first feeding time of the formula milk and complementary food.The total score of the ICFI was obtained by summing the scores of the indicators according to the 6- to 8-month-old, 9 to 11-month-old and 12 to 24-month-old categories.The total score of the ICFI evaluation index used in this study was 15 points. According to the Chinese Center for Disease Control, ICFI above 9 points was classified as qualified.

### Sample size estimation

The sample size for the reliability and validity test is 5–10 times the number of "subscale" entries containing the most items in the questionnaire [19]. In this FFQ, the maximum number of entries in the questionnaire is 9 and increased by 10 times, that means the sample size is not less than 90.

In this survey, a convenient sampling method was used to conduct the reliability and validity tests. First, number the overall survey units one by one; arbitrarily specify the starting point and order of sampling on the random number list; then draw sample unit numbers from the random number list in turn. Any number drawn within the number range is the number of the sample unit, until the number is full.Finally, 100 persons were randomly selected from nearly 300 infants and young children aged 7–24 months,and after inclusion and exclusion, the final sample size for the reliability test was 95 and the final sample size for the validity test was 98.

### FFQ and 24 h DRs

The 79-food-items FFQ of 7–24 months old infants and young children was revised based on the 2016 Chinese Dietary Guidelines, the feeding guidelines for infants and young children aged 7–24 months, and the previous FFQs in China, taking into account the dietary feeding characteristics of infants and young children [20]. The dietary questionnaire included the frequency of breastfeeding and formula feeding, the amount of formula milk every time (mL), the time of first supplementation of complementary food and formula, the frequency of complementary food supplements in the past week, the frequency and amount of fruit, vegetables, meat (chicken, duck, goose), red meat (pork, beef, lamb), fish, soy and soy products (soybean, soy milk, tofu, soy milk), solid liquid soy products, coarse grains (corn, millet, sweet potato, mung bean, sorghum, oats, buckwheat, etc.) and egg. The frequency of addition of each complementary food is divided into five categories: never, 2–3 times a month, 2–3 times a week, 4–5 times a week, 1 time or more per day.

The 24 h DRs were conducted using the multiple-pass method, developed by the US Department of Agriculture [21]. The 24 h DRs consist of precisely recalling, describing and quantifying the intake of foods and beverages consumed in the 24 h period prior to, or during the day before the interview, from the first intake in the morning until the last foods or beverages consumed at night (before going to bed or later, in the case of those who get up at midnight and eat and/or drink something). The information should describe the type of food and its characteristics (fresh, precooked, frozen, canned, preserved),the net quantity consumed, method of preparation, commercial brands, sauces, dressings (type of fats and oils used),condiments, liquids, multivitamin supplements and food supplements, as well as the time and place of consumption (at home, away from home), etc. [22].

### Statistical analysis

All analysis were conducted by IBM SPSS software version 25.0 (SPSS, IBM, New York, USA). In the reliability test, the correlation analysis was performed after encoding the frequency data of each level, such as frequency of complementary food supplement: 0 for "never"0, 1 for "2–3 times per month", 2 for "2–3 times per week", 3 for "4–5 times a week", and 4 for "1 or more times a day". In the validity test, the results of 24-h DRs were first coded by food classification and portion size conversion, then summed and averaged to the weekly frequency of food addition and the amount of each addition (number of servings or ml) in the same dimension as that of the FFQ; finally, the results of the FFQ and the converted 3-day 24-h DR results were coded uniformly: "never" was coded as 0 (0 times a week), "2–3 per month" was coded as 1 (one time per week), "2–3 times per week" was coded as 3 (3 times a week), and "4–5 times per week" was coded as 5 (5 times a week), "1 or more times per day” was coded as 7 (7 times a week), and the 3-day 24-h DR results were coded as integer parts of the weekly frequency.

Continuous variables are expressed as mean (Mean), median (Median) and percentile (P25, P75). The reliability test indicator is expressed by the interclass correlation coefficient (ICC) to measure the test–retest reliability, the value of the ICC is between 0–1, ICC < 0.4 indicates poor reliability, ICC > 0.75 indicates good reliability. In the validity test, the Spearman correlation coefficient was used as the index of validity test, with values ranging from -1 to 1. *ρ* > 0.7, *ρ* = 0.3–0.7, *ρ* < 0.3 separately means strongly correlated, moderately correlated, and weakly correlated, respectively, α < 0.05 [23]. The total score of the ICFI in this study is 15 and the ICFI ≥ 9 is qualified according to Chinese center for disease control [17].

## Result

### Reliability of the FFQ1 vs. FFQ2

The results of the test–retest reliability of FFQ1 *vs.* FFQ2 (Table 1) showed that the frequency and the portions of dietary supplements in the 7–10 days were all positively correlated. The average ICC was 0.536,ranged between 0.266 for portion of red meat and 0.904 for frequency of breastfeeding. Among them, the addition of breast milk, formula and the frequency of complementary food supplements had good reliability with ICCs were all above 0.5, the reliability ICCs for portions of various complementary foods were lower than the frequency of additions. The reliability of ICCs were all statistically significant (*P* < 0.05).

**Table 1:** Reliability of intakes of food groups between the FFQ1 and FFQ2 ^a^

Food groups	FFQ1				FFQ2				ICC	*P*-value
	Mean	Median	P25	P75	Mean	Median	P25	P75		
Frequency of breastfeeding (times/day)	2.4	0.0	0.0	5.0	2.2	0.0	0.0	5.0	0.904	< 0.001
Frequency of formula milk (times/day)	3.8	4.0	2.0	6.0	3.7	4.0	2.0	5.8	0.847	< 0.001
Formula milk (mL/per time)	105	120	50	150	103	120	60	150	0.581	< 0.001
Frequency of complementary food (times/day)	2.7	3.0	2.0	3.0	2.9	3.0	2.0	3.0	0.512	< 0.001
Frequency of fruit	2.2	2.0	1.0	4.0	1.9	2.0	1.0	3.0	0.563	< 0.001
Portion of fruit ^b^	0.6	0.5	0.5	1.0	0.6	0.5	0.5	0.5	0.301	0.002
Frequency of vegetables	1.9	2.0	0.0	3.0	1.7	2.0	1.0	2.0	0.720	< 0.001
Portion of vegetables ^b^	0.4	0.5	0.0	0.5	0.4	0.5	0.0	0.5	0.387	< 0.001
Frequency of meat	1.3	1.0	0.0	2.0	1.6	1.0	0.0	3.0	0.546	< 0.001
Portion of meat ^b^	0.3	0.0	0.0	0.5	0.5	0.5	0.0	0.5	0.339	< 0.001
Frequency of fish	1.3	1.0	0.0	2.0	1.3	1.0	0.0	2.0	0.688	< 0.001
Portion of fish ^b^	0.5	0.5	0.0	0.5	0.5	0.5	0.0	0.5	0.378	< 0.001
Frequency of red meat	2.0	2.0	0.0	2.0	2.0	2.0	0.0	3.0	0.736	< 0.001
Portion of red meat ^b^	0.4	0.5	0.0	0.5	0.6	0.5	0.0	1.0	0.266	0.004
Portion of soy products ^b^	0.4	0.0	0.0	0.0	0.5	0.0	0.0	1.0	0.588	< 0.001
Portion of solid soy products ^b^	0.1	0.0	0.0	0.0	0.2	0.0	0.0	0.5	0.515	< 0.001
Portion of liquid soy products ^b^	0.0	0.0	0.0	0.0	0.1	0.0	0.0	0.0	0.289	0.001
Frequency of coarse food grain	1.0	1.0	0.0	2.0	1.1	1.0	0.0	2.0	0.600	< 0.001
Portion of coarse food grain ^b^	0.4	0.5	0.0	1.0	0.5	0.5	0.0	1.0	0.371	< 0.001
Frequency of egg	1.7	2.0	0.0	2.0	1.6	2.0	0.0	2.0	0.655	< 0.001
Portion of egg ^b^	0.5	0.5	0.0	1.0	0.5	0.5	0.0	1.0	0.439	< 0.001

Abbreviation: *FFQ* food frequency questionnaire

FFQ for Chinese children aged 7–24 months with CHD.*n* = 95

^a^ FFQ1: the first time to complete the FFQ; FFQ2: the second time to complete the FFQ in the 7–10 days

^b^ Portion in the table means specific weight per serving: fruit, vegetable:100 g/portion; Meat (Chicken, duck, goose meat, etc.), red meat (Pork, beef and mutton): 40-50 g/portion; Egg: 1/portion; Soy products (soybean, soy milk, bean curd, soy milk), solid soy products: (1 portion = 20 g soybean = 45 g bean curd); liquid soy products: (1 bottle = 200 ml soy milk), coarse food grain (corn, millet, sweet potato, mung bean, sorghum, oats, buckwheat, etc.): 50-60 g/portion; beverages: 250 ml/portion

### Validity of the FFQ vs. 24 h DRs

The frequency of meal additions and portion recorded in the FFQ and 24 h DRs did not obey the normal distribution, so spearman-related analyses were used. The results of the validity test (Table 2) showed that the FFQ was positively correlated with the frequency and portion size of meal additions recorded in the 24 h DRs, and the average SCC was 0.318(range: 0.166- 0.856),60% SCCs were above 0.3.

**Table 2:** Validity of intakes of food groups between 24 h DRs and FFQ

Food groups	FFQ				24 h DRs				r_s_^a^	*P*-value
	Mean	Median	P25	P75	Mean	Median	P25	P75		
Frequency of breastfeeding (times/day)	5.0	0.0	0.0	11.7	11.2	0.0	0.0	25.7	0.856	< 0.001
Formula milk (mL/per time)	110	110	75	150	100	108	70	150	0.847	< 0.001
Frequency of complementary food (times/day)	17.5	14.0	14.0	14.0	25.0	23.3	18.7	30.3	0.497	< 0.001
Frequency of fruit	3.0	3.0	1.0	5.0	3.1	2.0	0.0	7.0	0.56	< 0.001
Portion of fruit	0.5	0.5	0.0	1.0	0.8	0.2	0.0	0.7	0.321	0.001
Frequency of vegetables	2.5	3.0	0.0	3.0	3.3	2.0	0.0	7.0	0.343	0.001
Portion of vegetables	0.4	0.5	0.0	0.5	0.3	0.2	0.0	0.5	0.231	0.022
Frequency of meat	1.4	0.0	0.0	3.0	0.8	0.0	0.0	0.0	0.166	0.103
Portion of meat	0.3	0.0	0.0	0.5	0.1	0.0	0.0	0.0	0.222	0.028
Frequency of fish	1.7	1.0	0.0	3.0	1.5	0.0	0.0	2.0	0.335	0.001
Portion of fish	0.6	0.5	0.0	1.0	0.3	0.0	0.0	0.5	0.354	< 0.001
Frequency of red meat	2.8	3.0	0.0	5.0	4.6	7.0	2.0	7.0	0.41	< 0.001
Portion of red meat	0.5	0.5	0.0	0.5	0.5	0.4	0.1	0.6	0.235	< 0.001
Portion of soy products	0.4	0.0	0.0	1.0	0.1	0.0	0.0	0.0	0.153	< 0.001
Portion of solid soy products	0.1	0.0	0.0	0.0	0.0	0.0	0.0	0.0	0.000	——
Portion of liquid soy products	0.1	0.0	0.0	0.0	0.0	0.0	0.0	0.0	0.345	< 0.001
Frequency of coarse food grain	1.2	1.0	0.0	1.0	1.6	0.0	0.0	2.0	0.215	0.034
Portion of coarse food grain	0.4	0.5	0.0	0.5	0.3	0.0	0.0	0.5	0.24	0.017
Frequency of egg	2.2	1.0	0.0	2.5	1.8	0.0	0.0	5.0	0.472	< 0.001
Portion of egg	0.5	0.5	0.0	1.0	0.4	0.0	0.0	1.0	0.369	< 0.001

Abbreviation: *FFQ* food frequency questionnaire. *24 h DRs* 24-h diet records from 3 consecutive days

FFQ and 24 h DRs for Chinese children aged 7–24 months with CHD.*n* = 98

Referring to Table 1

^a^ Spearman correlation analysis was adopted because the frequency and portion of food in FFQ and the 24 h DRs records were not subject to normal distribution

Among them, the frequency of breastfeeding and formula milk had good validity (SCC > 0.5), so did the frequency of fruits, red meat, egg (SCC > 0.4), however, the frequency and portion of the supplementary food had a low value of SCC. The frequency and portion of solid soy products were almost 0 so the index of validity could not be drawn. Except for the frequency of meat, the SCCs of the remaining validity indicators were statistically significant (*P* < 0.05).

### Infant and child feeding index

The ICFI was 7.68 ± 3.69 in the samples tested for reliability and validity, and the acceptability of the ICFI was 39.9% (77/193).

The reliability and validity of the ICFI were separately calculated for all samples, and the results (Table 3) showed that the reliability ICCs were good (range: 0.374–0.958), and were all statistically significant(*P* < 0.01); the validity SCCs ranged between -0.066 for frequency of eggs and 0.834 for breastfeeding, the SCC of each item was less than 0.4 except for breastfeeding and nipple use, among this,only frequency of fruits (times/week) and 24 h complementary food species were statistically significant (*P* < 0.05).

**Table 3:** Reliability and validity of each items and total index of the ICFI

Food groups	Reliability		Validity	
	ICC	*P*-value	r_s_^a^	*P*-value
Breastfeeding or not	0.958	< 0.001	0.834	< 0.001
Use a nipple or not	0.752	< 0.001	0.706	< 0.001
Frequency of complementary food (times/24 h)	0.374	< 0.001	0.055	0.590
Frequency of fruits (times/week)	0.481	< 0.001	0.326	0.012
Frequency of vegetables (times/week)	0.622	< 0.001	0.084	0.413
Frequency of meat, fish and shrimp (times/week)	0.706	< 0.001	0.043	0.675
Frequency of beans (days/week)	0.546	< 0.001	0.059	0.566
Frequency of coarse food grain (days/week)	1.000	< 0.001	0.066	0.524
Frequency of eggs (days/week)	0.604	< 0.001	-0.066	0.523
24 h complementary food species	Na	Na	0.543	< 0.001
Dairy and dairy products	Na	Na	Na	Na
Time to add formula milk	Na	Na	Na	Na
Time to add complementary food	0.767	< 0.001	Na	Na
ICFI (missing four items)	0.705	< 0.001	0.391	< 0.001

Abbreviation: *ICFI* Infant and Child Feeding Index

Na: items which cannot be calculated by the questionnaire data

^a^ Spearman correlation coefficient

## Discussion

This study evaluated the reliability and validity of the FFQ and ICFI for the infants and young children of 7–24 months after cardiac surgery for congenital heart disease in Guangzhou from July to October 2018. The current situation of the ICFI and its qualification rate were described. Our result demonstrated a satisfactory reliability and moderate validity for all food groups intakes. The reliability of the ICFI is acceptable, but the validity results are quite different, indicating that the questionnaire is limited in the evaluation of the ICFI.

The reliability test index ICC in this study was between 0.266–0.904, with an average of 0.536. Compared with the local and foreign studies, the ICC was higher than other studies [10, 24], indicating that the reliability of the questionnaire was acceptable. The result showed the reliability of breast milk and formula addition was the best, which is primarily due to that the breast milk and formula milk are the main food resources of infants and young children aged 7–24 months. The reliability of the complementary food’s portion was not as good as its frequency, because the frequency is high and stable, but the portion may be varied with the recent climate, family eating habits and preferences, etc.. It is difficult to get statistically relevant results of the beverage while 7–24 months old infants and young children drink less beverage and the Chinese infant and young children's dietary guidelines also recommend adding dairy products after 12 months old [25]. In addition, the reliability of the questionnaire may also be affected by other factors. For instance, the duration of the time interval would affect the reliability of the questionnaire, so we adopted a 7–10 days interval consistently. Secondly, the investigator can also produce certain deviation values due to recall bias or curiosity [26].

The validity test index SCC in this study was between 0.166 and 0.856, with an average value of 0.318 and 60% SCC was above 0.3. Similar to local and foreign researches [10, 24], the validity of the questionnaire was acceptable. Among them, the breast milk and formula addition had good validity(SCC > 0.5), which may be related to the higher frequency of daily addition. The frequency of feedings in the 24 h DRs was significantly higher than that of the FFQ, because the frequency of feedings is unstable and easy to be affected by the infant's own condition. Among the frequency of complementary food supplements, the fruit, red meat and eggs were better reflected in the 24 h DRs and had a higher degree of coincidence with the FFQ, with SCC were all above 0.4, considering they were the most common food intake for infants and family members [27]. The SCC of the portion of complementary food supplement was below 0.37, mainly because the 24 h DRs was not comprehensively reflecting the supplement of one-week complementary food supplement. Compared with the recalled FFQ, the 24 h DRs cannot comprehensively reflect the addition of various complementary foods for infants and toddlers in a week, which leads to a decrease in the validity, and the decrease extent of portion was greater than the frequency. In addition, it also may be that most infants were 9–11 months old, infants who at this stage have less teeth germination so they could hardly eat tough food. The poultry meat was added less in the 24 h DRs (P25, P75 is 0, the mean is 0.1), which may lead to its SCC is not statistically significant [25].

This study has strengths and limitations that need to be described.The 24 h DRs which selected as the "gold standard" by the validity test of this study, reflecting the real-time specific dietary addition of the respondents in three day, and had smaller recall bias and stronger training effect [28, 29]. In addition, when comparing the specific frequency indicators of the two questionnaires, such as the frequency of complementary food supplements in a week, the FFQ needs to scale the corresponding options to one week, while the 24 h DRs needs estimate the supplement status of one week from the supplement status of three days, which may be resulted in the concentration of the variety and portion of the complementary food supplements. As a result, the information loss and the estimated deviation caused by the questionnaire comparison may result in weak correlation of the two questionnaires.

The score of the ICFI was 7.68 ± 3.69, which was higher than the average level of large cities in China, similar to the results of HanZhong in Shanxi and Chengdu [30]. The high score may be related to the good care of taken by the caregivers of the infants after congenital heart disease surgery. And the qualification rate was 39.9% (77/193), which was higher than the value of the national small and medium-sized cities, [31] indicating that the feeding mode of caregivers is acceptable. The reliability of the scores of each item in the questionnaire was good (range: 0.374–0.958), and the ICC score of 80% items was above 0.5, which was consistent with the reliability study of the questionnaire, indicating that the questionnaire had a good consistency of the ICFI scores. The validity index SCC was quite different (range:-0.066 to 0.834), the SCC of the breastfeeding and pacifier use were quite well which may be due to the higher frequency, followed by the frequency of fruit addition. The lower validity of other supplementary food addition items may be due to the conversion result caused by difference of the questionnaires or the difference in frequency and portion between the two questionnaires results.

The subjects of this study were only selected from infants and young children aged 7–24 months in Guangzhou in August to October 2018. Secondly, for the selection of "gold standard", the 24 h DRs is the most common, but there are certain limitations for reflecting the addition of one week’s complementary food, when the recording time is extended or other dietary survey methods are combined to test the validity. Thirdly, the items and evaluation indicators for the ICFI have not been fully unified. Fourthly, no validation with biomarkers is carried out. Finally, ten days to evaluate the reliability is a very short period and it might have had an influence in the high correlation for some groups.

## Conclusion

The FFQ possesses satisfactory reliability and moderate validity. The reliability of the ICFI is acceptable, but the validity results are quite different, indicating that the questionnaire is limited in the evaluation of the ICFI.

## Supplementary Information

**Additional file 1: Supplementary file 1. **Congenital Heart Disease Patients Nutrition and Growth Questionnaire (7-24months old).

**Additional file 2: Supplementary file 2. **24 hour dietary recall.

## Data Availability

The raw data is available through this website: https://rdd.sysu.edu.cn/.

## Abbreviations

CHD: Congenital heart disease
FFQ: Food frequency questionnaire
ICC: Intraclass correlation coefficients
ICFI: Infant and child feeding index
SCC: Spearman correlation coefficient
24 h DRs: 24-H diet records for 3 consecutive days
