# Supplementary material for: Reliability and validity of the FFQ and feeding index for 7-to 24-month-old children after congenital heart disease surgery
Source: BMC Pediatr. 2022 Jun 16;22:348. doi: 10.1186/s12887-022-03357-4 (PMC9202103; doi:10.1186/s12887-022-03357-4)
Supplement: Supplementary file 1 — Additional file 1: Supplementary file 1. Congenital Heart Disease Patients Nutrition and Growth Questionnaire (7-24months old). [file 12887_2022_3357_MOESM1_ESM.doc]

# Congenital Heart Disease Patients Nutrition and Growth Questionnaire

# （7-24months old）

Dear parents,

The present questionnaire was designed to investigate your child's nutritional status and its impact on heart and growth. The questionnaire was mainly about your child's growth, **Dietary nutrition**, and lifestyle. There is no right or wrong answer, please fill in the questionnaire according to your child's real situation. We promise you that the information you fill in is completely confidential.

The questions in the questionnaire are all **single-choice** unless it is specified. Please put a **“√”** before the number of the option that matches the situation. Eevery question is important, **so please don't miss any of it**! Completing questionnaire will take you approximately 10 minutes.

(*Copyright: The contents of this questionnaire were designed by Associate Professor Yanna Zhu, School of Public Health, Sun Yat-sen University. If anyone wants to reprint or use, please contact zhuyn3@mail.sysu.edu.cn )

Women and Children’s Medical Center of Guangzhou

School of Public Health, Sun Yat-sen University

November, 2017

Child’s name: gender: ______ Date of Birth: Day Month Year

Address: ____________________________ Parents Phone Number: _________________ Wechat Number: Date of filling: Day Month Year

Health Care Handbook Number: _________ Outpatient Number: _____________________

**Part 1. General information**

1. Preparer:

[1]Father [2]Mother [3]Both father and mother [4]Grandparents [5]Other

1. Your child’s ***current*** weight is： kg(Kilogram); height： cm(centimeter);

Head circumference：______cm; Chest circumference：______cm；

3. Generally speaking, the health status of your child is: ；

[1]very poor [2]poor [3]fair [4]good [5]very good

4. Has your child been diagnosed with any of the following disease ***in the past 6 months?***

| Disease | Diagnosed or not | Age when illness first appeared |  | Disease | Diagnosed or not | Age when illness first appeared |
| --- | --- | --- | --- | --- | --- | --- |
| Pneumonia | [1] Yes  [2] No | months old |  | Febrile convulsions | [1] Yes  [2] No | months old |
| Heart Disease | [1] Yes  [2] No | months old |  | Diabetes | [1] Yes  [2] No | months old |
| Thalassemia | [1] Yes  [2] No | months old |  | Iron deficiency anemia | [1] Yes  [2] No | months old |
| Asthma | [1] Yes  [2] No | months old |  | Eczema | [1] Yes  [2] No | months old |
| Allergic dermatitis | [1] Yes  [2] No | months old |  | Urticaria | [1] Yes  [2] No | months old |
| Anaphylactic rhinitis | [1] Yes  [2] No | months old |  | Rickets | [1] Yes  [2] No | months old |
| Pathological jaundice | [1] Yes  [2] No | months old |  | Other（____） | [1] Yes  [2] No | months old |

5. Has your child been diagnosed with any of the following diseases ***in the past 6 months***?（Multiple choices available）？

| Disease | Diagnosed or not | Times of disease | Disease last longer than 3 days or not |
| --- | --- | --- | --- |
| Cold | [1] Yes [2] No | Times | [1] Yes [2] No |
| Bronchitis | [1] Yes [2] No | Times | [1] Yes [2] No |
| Diarrhea | [1] Yes [2] No | Times | [1] Yes [2] No |
| vomiting | [1] Yes [2] No | Times | [1] Yes [2] No |
| Constipation | [1] Yes [2] No | Times | [1] Yes [2] No |
| Thrush | [1] Yes [2] No | Times | [1] Yes [2] No |

**Part 2. Feeding pattern**

1. Which of the following feeding practices did you choose ***now***? (breast feeding and/ or formula feeding)

[1] Breastfeeding [2] mixed feeding（breastfeeding + formula） [3] formula feeding

If it was exclusive breastfeeding, it was given to your child ____ times/ day in the past 3 days；

If it was adopted, it happened when your child was ____ months of age.

2. Have you started adding formula milk or dairy substitutes to your child's diet：

[1] Yes [2] No (If the answer is “No”, please skip to question 5.)

（1）The formula milk or dairy substitutes was first added when your child was____ months of age, and the product is: (Multiple choices available)

[1] Preterm formula milk [2] Regular formula milk

[3] Anti-allergy formula milk（Hydrolyzed protein milk, etc）

（2）The brand of your formula milk or dairy substitutes was: ____________

（3）The reason of adding formula milk or dairy substitutes was: (Multiple choices available)

[1] No breast milk [2] Lack of breast milk [3] Mother refused breast feeding

[4] Family didn’t support breastfeeding [5] Can’t provide breastfeeding due to work [6] Mother was not around her child [7] Other

（4）In the past 3 days, your child was fed with formula milk ____ times/day, and ___ ml for each time.

3. Has your child started to have complementary foods :

[1] Yes [2] No (If the answer is “No”, please skip to question 7.)

4. The complementary foods were started when your child was____ months of age;

In the past 7 days, your child has complementary foods ____ times/day;

Please fill in the first addition time (child’s months of age) and food names of the following 11 kinds of complementary foods

| 1) Cereals and potatoes (Rice paste, porridge, rice, noodles, mashed potatoes, etc)： | First added at ____months of age; | Food name:________ |
| --- | --- | --- |
| 2) Vegetables: | First added at ____months of age; | Food name:________ |
| 3) Fruits: | First added at ____months of age; | Food name:________ |
| 4)Eggs(egg folk/egg white/full egg): | First added at ____months of age; | Food name:________ |
| 5) Aquatic Products (fish, etc): | First added at ____months of age; | Food name:________ |
| 6) Poultry meat（Chickens, ducks, geese, etc.）: | First added at ____months of age; | Food name:________ |
| 7) Livestock meat（Pork, beef, mutton, etc.）: | First added at ____months of age; | Food name:________ |
| 8) Nuts (Walnuts, peanuts, etc.): | First added at ____months of age; | Food name:________ |
| 9) Salt: | First added at ____months of age; | Food name:________ |
| 10)Sugar water（Water mixed with white sugar or brown sugar or honey, etc.）: | First added at ____months of age; | Food name:________ |
| 11）Soybeans (soybeans, soy milk, tofu, soy milk): | First added at ____months of age; | Food name:________ |

5.（1）Do you think your child has a regular diet?

[1]Regular time and quantity [2]Regular time but not regular quantity

[3]Regular quantity but not regular time [4] No regular time and quantity

（2）***In the last 30 days***, did you feed your child at night? [1]Yes [2]No

（3）***In the last 30 days,*** your child ate most at____, and least at____ in a day.

[1] breakfast [2]lunch [3]dinner

（4）***In the last 30 days***，was your child a picky eater? [1]Yes [2]No

（5）***In the last 30 days***, your child’s favorite food was___, and most disliked food was___.(Multiple choices available)

[1] rice [2] meat [3] fish, shrimps [4] eggs [5] vegetables [6] fruits

[7] soybeans and soy food [8] milk and dairy products [9] animal viscera

[10] pickled vegetables

1. Your child’s dietary taste prefer:

[1] Salty [2] Fair [3] Light (less salt)

(7) Generally speaking, your child’s appetite of ***the last 30 days*** was:

[1] very poor [2] poor [3] fair [4] good [5] very good

(8) ***In the last 30 days,*** were there any difficulties in feeding?

[1] Yes [2] No（If the answer is “NO”, please skip to question 9）

If the answer is “Yes”, what kind of difficulties was it? (Multiple choices available)]

[1] Your child was a picky eater [2] Your child did not eat at the regular time

[3] Your child had a bad appetite [4] Your child suffered from diseases

[5] Your child was noisy [6] It was too tired to feed your child

[7] Lack of feeding experience [8] Others___

(9) Do your child concentrate during meals? [1] Yes [2] No

(10) Do your child watch TV or use other electronic devices during meals? [1] Yes [2] No

(11) Now, your child’s average time of a meal is____ minutes.

6. In the last 30 days, how often did your child eat fruit?_


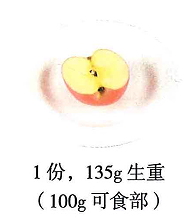

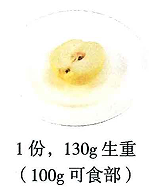
 [1] Never；[2] 2-3 times a month；[3] 2-3 times a week；[4] 4-5 times a week； [5]once a day or more；

How many servings of fruit did your child eat for a time on average? _____servings（1 serving=100g, as shown below）

7. In the last 30 days, how often did your children eat vegetables? ____

[1] Never；[2] 2-3 times a month；[3]2-3 times a week；[4]4-5 times a week； [5]once a day or more；


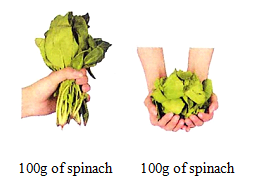
How many servings of vegetables did your child eat for a time on average? ________servings（1 serving=100g, as shown below）

8. In the last 30 days, how often did your children eat pork? ____


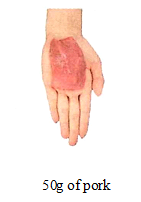
[1] Never；[2] 2-3 times a month；[3]2-3 times a week；[4]4-5 times a week； [5]once a day or more；

How many servings of pork did your child eat for a time on average? ________servings（1serving=40-50g, around size of a palm, as shown below）

9. In the last 30 days, how often did your children eat fish? ____


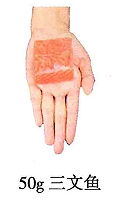
[1]Never；[2] 2-3 times a month；[3]2-3 times a week；[4]4-5 times a week； [5]once a day or more；

How many servings of fish did your child eat for a time on average?

________servings（1serving=40-50g, around size of a palm, as shown below）

10. In the last 30 days, how often did your children eat red meat (pork and mutton)? ____


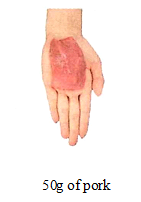
[1]Never；[2] 2-3 times a month；[3]2-3 times a week；[4]4-5 times a week； [5]once a day or more；

How many servings of red meat did your child eat for a time on average? ________servings （1serving=40-50g, around size of a palm, as shown below）

11. In the last 30 days, how often did your children eat soybean and its products? ____

[1] Never；[2] 2-3 times a month；[3]2-3 times a week；[4]4-5 times a week； [5]once a day or more；


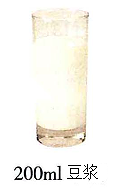

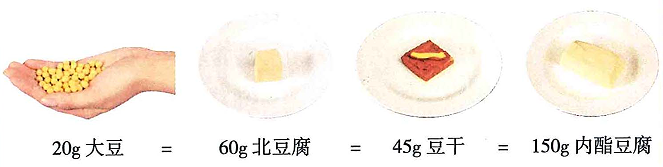
How many servings of soybean and its products did your child eat for a time on average? ________servings. （1serving=20g of soybean=60 g of tofu= 45 g of dried tofu, 1 cup of liquid bean products=200 ml soybean milk, as shown below）

12. In the last 30 days, how often did your children eat coarse food grain? ____

[1] Never；[2] 2-3 times a month；[3]2-3 times a week；[4]4-5 times a week； [5]once a day or more；

How many servings of soybean and its products did your child eat for a time on average? ________servings（1serving=50-60 g, around size of an egg）

13. In the last 30 days, how often did your children eat eggs (hen’s egg)? ____

[1]Never；[2] 2-3 times a month；[3]2-3 times a week；[4]4-5 times a week； [5]once a day or more；

How many eggs did your child eat for a time on average? ________servings.

（1egg=50-60 g, around size of a hen’s egg, a hen’s egg= 3 pigeon eggs= 2 quail eggs= a half of a goose’s egg ）

14.（1）In the last 30 days, how often did your children drink milk beverages (such as sour milk beverage and milk drinks)?

[1]Never；[2] 2-3 times a month；[3]2-3 times a week；[4]4-5 times a week；


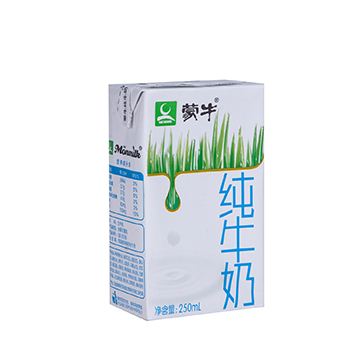
[5]once a day or more；

How many bottle of milk beverages did your child drink for a time on average? ________bottles (1bottle=250 ml).

（2）In the last 30 days, how often did your children drink sugar-sweetened beverages (such as soft drink, juice, tea drinks, coffee and sports drinks)?

[1]Never；[2] 2-3 times a month；[3]2-3 times a week；[4]4-5 times a week； [5]once a day or more；


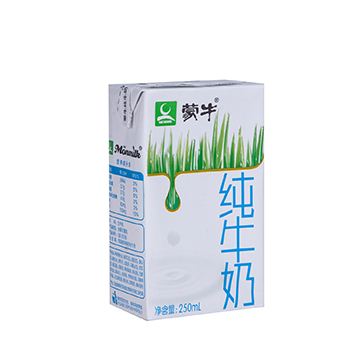
How many bottle of sugar-sweetened beverages did your child drink for a time on average? ________bottles (1bottle=250 ml).

15. In the last 30 days, how often did your children eat snacks (cookies, cakes and chocolates) ?

[1] Never；[2] 2-3 times a month；[3] 2-3 times a week；[4] 4-5 times a week； [5] once a day or more；

If your child has the habit of eating snacks, which three of the following are most eaten?（Pick 3 snacks which your child eat most）

[1] biscuits [2] cakes [3] candies [4] chocolate [5] dried meat slice [6] puffed food (such as potato chips) [7] other

16. Did your child have nutritional supplements？

[1]Yes [2]No (If answer is “No”, please skip to question 1 of Part.3 Life style )

If the answer is “Yes”, how was the detail of your child's vitamin and mineral supplementation in the last 30 days?

1. vitamin D：

[1] Never；[2] 2-3 times a week；[3] 4-6 times a week； [4] once a day

2）vitamin AD：

[1] Never；[2] 2-3 times a week；[3] 4-6 times a week； [4] once a day

3）Cod liver oil：

[1] Never；[2] 2-3 times a week；[3] 4-6 times a week； [4] once a day

4）Calcium：

[1] Never；[2] 2-3 times a week；[3] 4-6 times a week； [4] once a day

5）Iron：

[1] Never；[2] 2-3 times a week；[3] 4-6 times a week； [4] once a day

6）Zinc：

[1] Never；[2] 2-3 times a week；[3] 4-6 times a week； [4] once a day

7）Protein powder：

[1] Never；[2] 2-3 times a week；[3] 4-6 times a week； [4] once a day

（The dietary images in the questionnaire are from the 2016 edition of the Dietary Guidelines for Chinese Residents）

**This is the end of the questionnaire, thank you for your cooperation!**
