# Supplementary material for: Reliability and validity of the FFQ and feeding index for 7-to 24-month-old children after congenital heart disease surgery
Source: BMC Pediatr. 2022 Jun 16;22:348. doi: 10.1186/s12887-022-03357-4 (PMC9202103; doi:10.1186/s12887-022-03357-4)
Supplement: Supplementary file 2 — Additional file 2: Supplementary file 2. 24 hour dietary recall. [file 12887_2022_3357_MOESM2_ESM.docx]

**24 hour dietary recall**

**Instructions：**

1. Record dietary intake for 3 days, it should include 2 working days and 1 rest day, such as Thursday, Friday, Saturday, or Sunday, Monday, Tuesday. Please record 1 day dietary in a table and 3 tables in total.
2. All the food your child ate at home or out should be recorded, including breast milk, milk, complementary foods, water, drinks, etc.
3. Filling methods：

- Record the child’s every feeding in detail, including the amount of milk or complementary foods.
- The "Food name" should be specific, which should tell the ingredients or trademarks, such as: Mead Johnson milk, Jiabao rice paste, etc.
- The “Food composition" refers to the specific ingredients of the food, such as the "food composition" for celery and beef dumplings is celery, beef, and flour. Commercial food, such as cookies, and beverages, can be written without food composition.
- In "Food weight", if you are not sure how much the food weight, please compare to the following food chart for estimation (as shown in Chart 1), using "grams (g) or milliliters (ml)". For example, half an apple is about 135g.
- Complementary foods are all foods other than milk or breast milk, including rice paste, porridge, vegetables, fruits, eggs, and all kinds of meat. When filling in the form, please compare the amount of each food carefully to the food chart (as shown in Chart 1). Please refer to Table 1 for the filling method.

Chart 1. Standard food chart

| Cereals and potatoes | 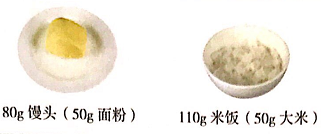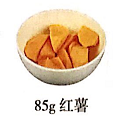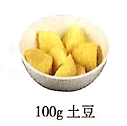 |
| --- | --- |
| Fruits | 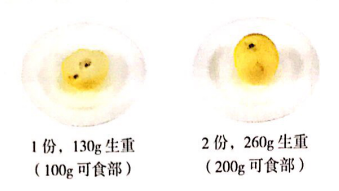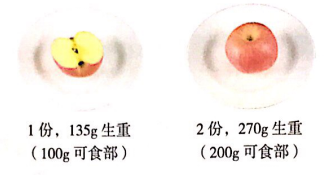 |
| Vegetables | 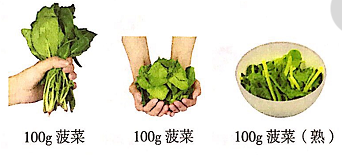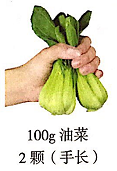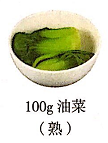 |
| Meats | 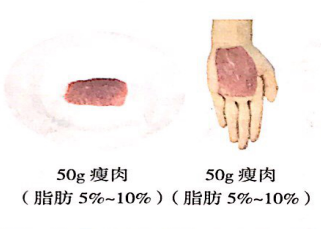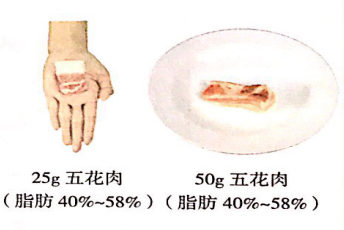 |
| Fish | 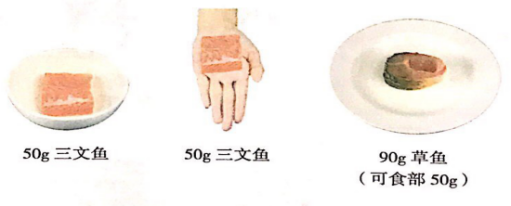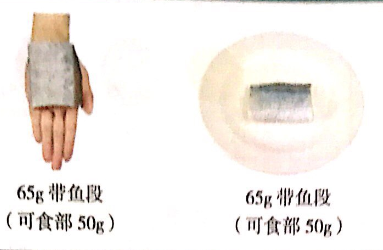 |
| Shrimps | 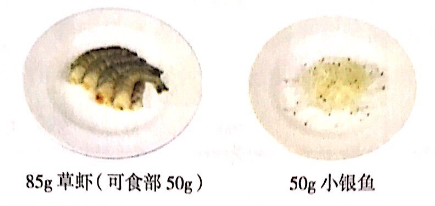 |
| Eggs | 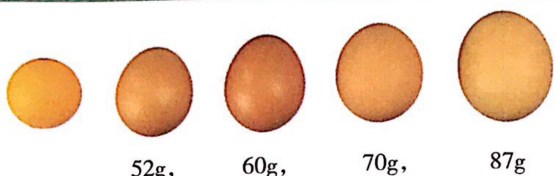 |
| Soy beans | 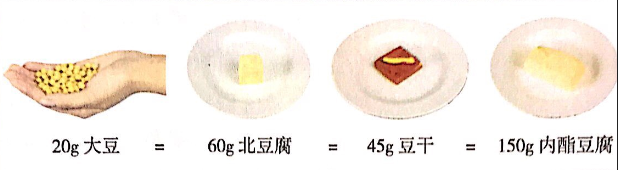 |
| Various container sizes | 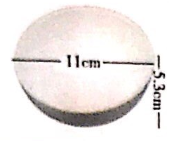 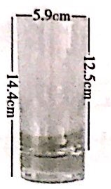 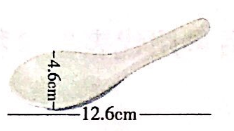  250ml 10ml |

Table 1 Example for the child’s dietary record

| Time | | Food name | Food composition | Serving | | Location |
| --- | --- | --- | --- | --- | --- | --- |
|  |  |  |  | Quantity and units | Weight |  |
| Moring | 7:00 | Bellamy milk | Milk powder | 5 spoons | 150ml | Home |
| Moring | 8:30 | Apple | Apple | Half of it | 135g | Home |
| Moring | 10:00 | Bellamy milk + Garbo rice paste | Milk powder， rice | Milk powder 3 spoons，rice paste 1 spoon | Milk 90ml，rice paste 22g | Home |
| Noon | 12:00 | Steamed turbot | fish | ¼ fish | 100g | Home |
| Afternoon | 14:00 | Bellamy milk | Milk powder | 5 spoons | 150ml | Home |
| Afternoon | 16:00 | Orange | Orange juice | ⅓ cups | 90ml | Home |
| Evening | 18:00 | Spinach and lean pork porridge | Spinach, lean pork, rice | 1 Spinach and 1 lean pork, and half dish of porridge | Spinach 50g，lean pork 50g，porridge 50g | Home |
| Evening | 21:00 | Bellamy milk | Milk powder | 5 Spoons | 150ml | Home |
| Night | 24:00 | Bellamy milk | Milk powder | 5 Spoons | 150ml | Home |
| Night | 4:00 | Bellamy milk | Milk powder | 5 Spoons | 150ml | Home |

**General information of child：**

Name： Sex： Age：

Height： （cm） Weight： （kg）

Has your baby been taking nutritional supplements (e.g., vitamin D3, vitamin AD drops, cod liver oil, calcium, iron, zinc, etc.) regularly since birth？ ①No ②Yes，regularly ③Yes, irregularly ④Do not know; If your answer is ② or③, please write down the name, brand and amount of the supplements :

**Dietary record table（a 24-hour record）: First day**

**Date of filling: ____ Day ____ Month ____ Year**

- Please check at the end of the day, whether all the food your child eaten is filled in the table, and don't forget the supplementary food!
- Please fill in the table carefully, and we will assess your child’s eating habits and intake of key nutrients.

| Time | | Food name | Food composition | Serving | | Location |
| --- | --- | --- | --- | --- | --- | --- |
|  |  |  |  | Quantity and units | Weight |  |
|  |  |  |  |  |  |  |
|  |  |  |  |  |  |  |
|  |  |  |  |  |  |  |
|  |  |  |  |  |  |  |
|  |  |  |  |  |  |  |
|  |  |  |  |  |  |  |
|  |  |  |  |  |  |  |
|  |  |  |  |  |  |  |
|  |  |  |  |  |  |  |
|  |  |  |  |  |  |  |
|  |  |  |  |  |  |  |
|  |  |  |  |  |  |  |
|  |  |  |  |  |  |  |
|  |  |  |  |  |  |  |
|  |  |  |  |  |  |  |
|  |  |  |  |  |  |  |
|  |  |  |  |  |  |  |
|  |  |  |  |  |  |  |

Today, your child's water intake is ____ ml, oil intake is _____ g, and salt intake is ____ g.

**Dietary record table（a 24-hour record）: Second day**

**Date of filling: ____ Day ____ Month ____ Year**

- Please check at the end of the day, whether all the food your child eaten is filled in the table, and don't forget the supplementary food!
- Please fill in the table carefully, and we will assess your child’s eating habits and intake of key nutrients.

| Time | | Food name | Food composition | Serving | | Location |
| --- | --- | --- | --- | --- | --- | --- |
|  |  |  |  | Quantity and units | Weight |  |
|  |  |  |  |  |  |  |
|  |  |  |  |  |  |  |
|  |  |  |  |  |  |  |
|  |  |  |  |  |  |  |
|  |  |  |  |  |  |  |
|  |  |  |  |  |  |  |
|  |  |  |  |  |  |  |
|  |  |  |  |  |  |  |
|  |  |  |  |  |  |  |
|  |  |  |  |  |  |  |
|  |  |  |  |  |  |  |
|  |  |  |  |  |  |  |
|  |  |  |  |  |  |  |
|  |  |  |  |  |  |  |
|  |  |  |  |  |  |  |
|  |  |  |  |  |  |  |
|  |  |  |  |  |  |  |
|  |  |  |  |  |  |  |

Today, your child's water intake is ____ ml, oil intake is _____ g, and salt intake is ____ g.

**Dietary record table（a 24-hour record）: Third day**

**Date of filling: ____ Day ____ Month ____ Year**

- Please check at the end of the day, whether all the food your child eaten is filled in the table, and don't forget the supplementary food!
- Please fill in the table carefully, and we will assess your child’s eating habits and intake of key nutrients.

| Time | | Food name | Food composition | Serving | | Location |
| --- | --- | --- | --- | --- | --- | --- |
|  |  |  |  | Quantity and units | Weight |  |
|  |  |  |  |  |  |  |
|  |  |  |  |  |  |  |
|  |  |  |  |  |  |  |
|  |  |  |  |  |  |  |
|  |  |  |  |  |  |  |
|  |  |  |  |  |  |  |
|  |  |  |  |  |  |  |
|  |  |  |  |  |  |  |
|  |  |  |  |  |  |  |
|  |  |  |  |  |  |  |
|  |  |  |  |  |  |  |
|  |  |  |  |  |  |  |
|  |  |  |  |  |  |  |
|  |  |  |  |  |  |  |
|  |  |  |  |  |  |  |
|  |  |  |  |  |  |  |
|  |  |  |  |  |  |  |
|  |  |  |  |  |  |  |

Today, your child's water intake is ____ ml, oil intake is _____ g, and salt intake is ____ g.
